# 16 kDa Heat Shock Protein from Heat-Inactivated *Mycobacterium tuberculosis* Is a Homodimer – Suitability for Diagnostic Applications with Specific Llama VHH Monoclonals

**DOI:** 10.1371/journal.pone.0064040

**Published:** 2013-05-30

**Authors:** Saurabh K. Srivastava, Vincent J. B. Ruigrok, Natalie J. Thompson, Anke K. Trilling, Albert J. R. Heck, Cees van Rijn, Jules Beekwilder, Maarten A. Jongsma

**Affiliations:** 1 Plant Research International, Wageningen, The Netherlands; 2 Laboratory of Organic Chemistry, Wageningen University and Research Centre, Wageningen, The Netherlands; 3 Laboratory of Microbiology, Wageningen University and Research Centre, Wageningen, The Netherlands; 4 Biomolecular Mass Spectrometry and Proteomics, Bijvoet Center for Biomolecular Research and Utrecht Institute for Phamaceutical Sciences, Utrecht University, Utrecht, The Netherlands; 5 Netherlands Proteomics Centre, Utrecht, The Netherlands; Bose Institute, India

## Abstract

**Background:**

The 16 kDa heat shock protein (HSP) is an immuno-dominant antigen, used in diagnosis of infectious *Mycobacterium tuberculosis* (*M.tb.*) causing tuberculosis (TB). Its use in serum-based diagnostics is limited, but for the direct identification of *M.tb.* bacteria in sputum or cultures it may represent a useful tool. Recently, a broad set of twelve 16 kDa specific heavy chain llama antibodies (VHH) has been isolated, and their utility for diagnostic applications was explored.

**Methodology/Principal Findings:**

To identify the epitopes recognized by the nine (randomly selected from a set of twelve 16 kDa specific VHH antibodies) distinct VHH antibodies, 14 overlapping linear epitopes (each 20 amino acid long) were characterized using direct and sandwich ELISA techniques. Seven out of 14 epitopes were recognized by 8 out of 9 VHH antibodies. The two highest affinity binders B-F10 and A-23 were found to bind distinct epitopes. Sandwich ELISA and SPR experiments showed that only B-F10 was suitable as secondary antibody with both B-F10 and A-23 as anchoring antibodies. To explain this behavior, the epitopes were matched to the putative 3D structure model. Electrospray ionization time-of-flight mass spectrometry and size exclusion chromatography were used to determine the higher order conformation. A homodimer model best explained the differential immunological reactivity of A-23 and B-F10 against heat-treated *M.tb.* lysates.

**Conclusions/Significance:**

The concentrations of secreted antigens of *M.tb.* in sputum are too low for immunological detection and existing kits are only used for identifying *M.tb.* in cultures. Here we describe how specific combinations of VHH domains could be used to detect the intracellular HSP antigen. Linked to methods of pre-concentrating *M.tb.* cells prior to lysis, HSP detection may enable the development of protein-based diagnostics of sputum samples and earlier diagnosis of diseases.

## Introduction

Tuberculosis (TB), caused by *Mycobacterium tuberculosis* (*M.tb.*), is one of the most prevalent and serious infectious diseases worldwide [1]. Each year ∼9.4 million new cases are reported with an estimated global mortality in 2010 of 1.4 million people [2]. With problems like multiple drug resistance (MDR), treatment of diagnosed TB cases is becoming more and more difficult and challenging [3]. TB cases are often intensified due to malnutrition and other allied infections that decrease body immunity, like HIV, especially in the developing parts of the world [4], [5]. Early detection of TB guarantees fast treatment and can offer much better prognosis. Therefore, development of techniques for early and accurate detection iscalled for [6], [7].

There are many different types of diagnostic assays available for detection of tuberculosis. Bacterial cultures are sensitive, but too time consuming, taking 2–3 weeks for detection under optimal conditions [8]. Microscopic identification of acid-fast bacilli in sputum smears is a fast technique but less sensitive then culture techniques, laboratory intensive and dependent on high concentrations of bacteria [8]. Nucleic acid amplification methods are fast, highly specific and sensitive, but they are technically complex, expensive and require skilled personnel with high quality standards for accurate performance [9]. Other immunological methods tend to detect secreted antigen protein from *M.tb.* Sensitivity and specificity of these methods are also too low to detect the antigens in sputum. As a result they are mainly used on cultures which takes a long time to grow, i.e. around 2–6 weeks [9]–[12].

TB is most prevalent in poor, remote areas of the world, where reliable DNA-based diagnostic procedures that need costly, advanced laboratory infrastructure and personnel are not available. This contrasts with antibody-dependent assays which are more easily implemented. The development of better antibodies is, therefore, a high priority, especially if they are less subject to generating false positives [13], [14] from cross reactivity with similar antigens of non-pathogenic *Mycobacterium* species.

Monoclonal VHH antibodies have recently gained considerable attention due to their unique physico-chemical stability [15] as well as low molecular weight of ∼15 kDa. Considering the limitations of existing diagnostics of TB, VHH antibodies can be utilized as tools for improvement of the existing immunological tests in detection of TB. They are 3–4 times smaller than conventional antibodies due to lack of light chains and removal of conserved domains. Furthermore, they can be produced at low cost in yeast or bacteria, and are easy to handle with long shelf life [16], [17].

Previously, we described the selection and preliminary characterization of a panel of 12 VHH antibodies against *M.tb.* [18]. Using ELISA and SPR techniques it was demonstrated that these VHH antibodies were specific for TB-causing mycobacteria and exclusively recognized the 16 kDa heat shock protein (HSP) [18], which is known to be a major contributor to the pathogenicity of the *M.Tb.* bacterium [19]. During latent phase, *M.tb. persists* inside macrophages due to the presence of the 16 kDa HSP protein, and it is also the most dominant protein in the extract of *M.tb.* [20]. The best VHH antibody showed a high affinity for HSP, with a dissociation constant (K_d_) of 4×10^−10^ M [18]. HSP is a cell-internal protein which is peripherally associated with the membrane. [21] For diagnostics purposes, it can be isolated in higher concentrations than secreted proteins by pre-concentrating the bacteria [18].

The main objective of this study was to characterize the binding of *M.tb.* HSP epitopes, protein and lysates to different VHH antibodies and to evaluate optimal capture-detection probe combinations. The information can be used to develop low cost, robust protein-based diagnostic platforms for TB based on these antibodies.

## Materials and Methods

### HSP Peptides and Proteins and VHH Proteins

**Figure 1:**
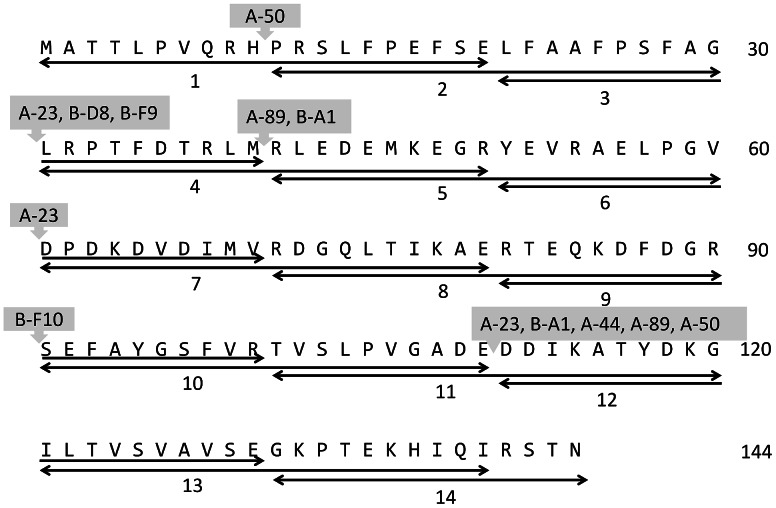
Summary of affinity interactions of 14 overlapping peptides derived from the HSP protein with 9 different anti-HSP VHH antibodies. VHH numbering is according to Trilling et al 2011 [18]. VHHs are indicated above the central portions of the interacting peptides.

Fourteen overlapping linear peptide epitopes (20 amino acid in length and with a 10 amino acid overlap; numbered 1–14, Figure 1) covering the entire HSP sequence (Figure S1) [22], [23], each covalently linked at the C-terminal with either an amide (-NH-R) group or biotin residue for direct and sandwich ELISA studies, were synthesized (Peptide 2.0 inc.). The synthesized peptides were purified by HPLC and their sequences were verified by mass spectrometry by the supplying company (Peptide 2.0 inc.). Two forms of VHH antibodies were used in this study, i.e. biotinylated VHH (VHH-AVI) and VHH with VSV tag (VHH-VSV). The production and purification of the these different VHH antibodies were done as described before [18] [24]. The four forms of HSP used in the present work were:

1. Recombinant HSP with AVI and HIS tag (rHSP-tag), produced and purified recombinantly from *E.coli* strain BL21 as described before [18].
2. Recombinant native HSP (rHSP) lysate, produced by PCR-amplifying the HSP from *M. tuberculosis* lysate using the primer HSP16.3-PstI-FW (5′- AAAAAAACTGCAGAAAATGGCCACCACCCTTCCC -3′) and HSP16.3- NotI- no.tag- RV (5′-TATATGCGGCCGCTTAGTTGGTGGACCGGATCTGA-3′) (PstI and NotI restriction sites are underlined). The digested fragment was inserted in a PstI-NotI cut PRI expression vector. This vector is based on the earlier described PRI-VSV expression vector [18]. The construct was transformed into *E. coli* XL-1 blue for multiplication. The cloned HSP sequence is identical to GenBank accession number S79751. Isolated plasmid DNA was transformed into *E. coli* strain BL21-AI for expression. Expression was performend as described before [18] and French press lysis was performed in native extraction buffer (150 mM NaCl, 5 mM imidazole, 20 µl DNase, 50 mM KH_2_PO_4_ at pH 8) supplemented with 1 mM PMSF and 2 µg/ml aprotinin and 1mg/ml lysozyme to final concentration.
3. Heated recombinant native HSP (heated-rHSP) lysate, production and expression of heated-rHSP was as described for rHSP. The only difference between the two samples was that, in case of heated-rHSP the cell pellet was heated for 30 min. at 80°C before French press.
4. HSP from *Mycobacterium tuberculosis* (*M.tb.*-HSP) lysate, produced and lysed as described before including a heat treatment as in 3 [18].

### ELISA-based Epitope Mapping of HSP

#### A. Direct ELISA

- *Linear epitopes as capture species*: Flat-bottom medium binding ELISA plates (Greiner Bio One) were coated via physical adsorption with 100 µl of each peptide at a concentration of 10^−4^ M in phosphate buffer saline (PBS) by incubating overnight at 4°C. Once these antigen-peptide coated wells were prepared, a standard ELISA method was followed as described by Trilling *et.al* [18] to determine the interacting epitopes of the 9 different VHH antibodies A-23, A-44, A-50, A-89, B-A1, B-B12, B-D8, B-F9 and B-F10.
- *VHH antibody as capture species:* ELISA plate wells (GreinerBioOne) were coated via physical adsorption with VHH antibodies (either A-23 or B-F10) as a capture element by incubating 100 µl of each antibody in PBS at a concentration of 2 µg/ml and incubated overnight at 4°C. These antibody-coated wells were washed with PBS and blocked using a blocking solution of 4 % non-fat powdered (w/v) milk in PBS (PBSM) for an hour. Later a mixture of 100 µl of each biotinylated peptide in PBS at a concentration of 10^−4^ M was added and allowed to interact for an hour. After incubating with antigen peptides, the wells were washed again with a solution of 200 µl of 0.05% TWEEN-20 in PBS (PBST). The wells were then subjected to streptavidin POD conjugate (Sigma Aldrich, USA) for an hour at a dilution of 1∶4000 in PBSM. Successive washing steps and measurements were done as described above. This procedure was applied to 6 different peptides (i.e. #1, #3, #5, #6, #9 and #11) which were selected on the basis of their response to VHH antibodies A-23, B-F10, A-44 and A-50.

#### B. Sandwich ELISA

Wells of ELISA plates were coated with VHH antibody A-23-AVI as capture element by incubating 100 µl of antibody in PBS at a concentration of 2 µg/ml and incubated overnight at 4°C, followed by an hour of blocking with PBSM at room temperature. Wells were then washed thrice with PBS solution followed by an hour of incubation with either rHSP-tag or *M.tb.*-HSP lysates, as described above. Plates were then blocked again for 1 hour with PBSM and washed three times with PBST. Then, the wells were incubated for an hour with the secondary sandwich VHH antibody A-23 or B-F10 with VSV tag, followed by three times washing with PBST and incubation for an hour with the detection antibody i.e. anti-VSV-HRP (Sigma Aldrich, Missouri, USA) before assay with 1-StepTM Ultra TMB substrate for ELISA (Pierce, IL).

### Size Exclusion Chromatography of HSP

For calibration of the column (Superdex 200 10/300 GL with length of 30 cm and diameter of 10 mm, GE life sciences), a mixture of known standards, i.e. Blue Dextran 2000, Ferritin, Catalase, Aldolase, Albumin, Ovalbumin, Chymotrypsinogen A and Ribonuclease A were first injected to obtain a calibration table (Supporting Information, Table S1, Figure S2). Once the column was calibrated, 250 µl of each HSP sample i.e. either rHSP-tag, rHSP, heated-rHSP with a concentration of 750 µg/ml or *M.tb.*-HSP lysates with concentration 450 µg/ml were loaded on to the column (at a flow of 0.75 ml/min, using PBS as running buffer). Fractions of 1 ml each were collected for further analysis. Diluted samples obtained from individual fractions were concentrated using the Amicon® Ultra centrifugal spin columns (Millipore Ireland Ltd. Ireland) with a cut off 10 kDa following the protocol supplied by the supplier. The concentrated fractions thus obtained were analyzed on dot blot. For dot blot, 100 µl of concentrated fractions were blotted in a circular spot with the help of SRC 96D S&S Minifold 1 dot blotter (Schleicher & Schuell, Germany) on nitrocellulose membrane (Trans-Blot, Bio-Rad, Hercules, CA) at room temperature. The membrane was then blocked with PBSM to avoid unspecific binding, washed with PBS and incubated with VHH antibody B-F10-VSV. A blocking step followed by washing and re-incubation of the membrane with anti-VSV-HRP antibody. Detection was done using 3,3,5,5′-Tetramethylbenzidine (TMB) liquid substrate system for membranes (Sigma-Aldrich, The Netherlands), by incubating the membrane for 10 min in the dark with TMB and then washing the substrate off with milliQ water and scanning the bands using a desktop scanner (Biorad GS-710).

### Electrospray Ionization Time-of-flight Mass Spectrometry

Electrospray ionization time-of-flight mass spectrometry (ESI-TOF-MS) is a native mass spectrometry method which is used to identify oligomerization of non-covalently associated protein oligomers [25], [26]. The method utilizes the soft ionization technique of nanoelectrospray ionization (nESI) to produce gas-phase ions, avoiding structural destruction of thermally labile large supramolecules, such as proteins and non-covalent protein complexes [27], [28]. The rHSP-tag sample was analyzed using nESI-TOF-MS to deduce the oligomerization after urea-based isolation. A small fraction of 20 µl of the sample was also retained for samples qualitative analysis on denaturing PAGE gel. The sample was first buffer-exchanged from PBS pH 9.0 to 150 mM ammonium acetate pH 9.0 using 5 kDa molecular weight cut off filter (Vivaspin 500, Sartorius Stedim Biotech GmbH, Goettingen, Germany). The protein was sprayed at a concentration of 20 µM on an ESI-TOF mass spectrometer (LCT, Waters, Manchester, UK) using gold-coated borosilicate capillaries made in-house (using a Sutter P-97 puller [Sutter Instruments Co., Novato, CA] and an Edwards Scancoat Six sputter-coater [Edwards Laboratories, Milpitas, CA]). Source backing pressure was increased to 6 mbar. Capillary voltage and cone voltage were set to 1300 and 200 V respectively. Mass calibration was performed using 25 mg/mL Cesium iodide. Data were analyzed using MassLynx V4.1 for experimental mass determination.

### Epitope Mapping Using Surface Plasmon Resonance

Surface plasmon resonance (SPR)-based sandwich experiments were performed using streptavidin-coated chips (GE Healthcare) in a Biacore 3000 system at 25°C, using HBS-EP buffer (pH 7.4, consisting of 10 mM 4-(2-hydroxyethyl) piperazine-1-ethane sulfonic acid, 150 mM sodium chloride, 3 mM ethylenediaminetetra acetic acid, 0.005% v/v surfactant polysorbate 20) as running buffer at a constant flow of 10 µl/min. The experimental setup was same as described before by Trilling *et al*. [18]. The chips had four independent channels out of which the first channel served as reference surface containing of VHH-M200 [18] that does not bind to the HSP (negative control), whereas the second and third channels contained VHH antibodies A-23 and B-F10, respectively. The fourth channel however was left untreated as a blank control. A total immobilization of 3000±100 RU was achieved with every antibody individually, used for anchoring purposes. Once the anchoring VHH antibodies were captured, all four channels were connected and subjected collectively to 50 µl of HSP at a concentration of 4 µg/ml. Binding yield of 2500 RU in channel 2 and 3. The chip was then subjected to 40 µl of secondary/sandwiching antibody VHH A-23 and the binding signals were recorded in terms on RU. The surface was regenerated using 10 µl of 10 mM hydrogen chloride solution (HCl). The process was then repeated by injecting HSP, followed by secondary/sandwiching VHH antibody B-F10. To verify the reproducibility, the whole experiment was repeated thrice.

## Results

### ELISA- and SPR-based Epitope Mapping of HSP

To study the region of the HSP protein responsible for interacting with VHH antibodies ELISA- and SPR-based epitope mapping techniques with HSP peptide overlapping epitopes and HSP intact protein were employed.

#### A. Epitope mapping using linear peptides in direct ELISA

**Figure 2:**
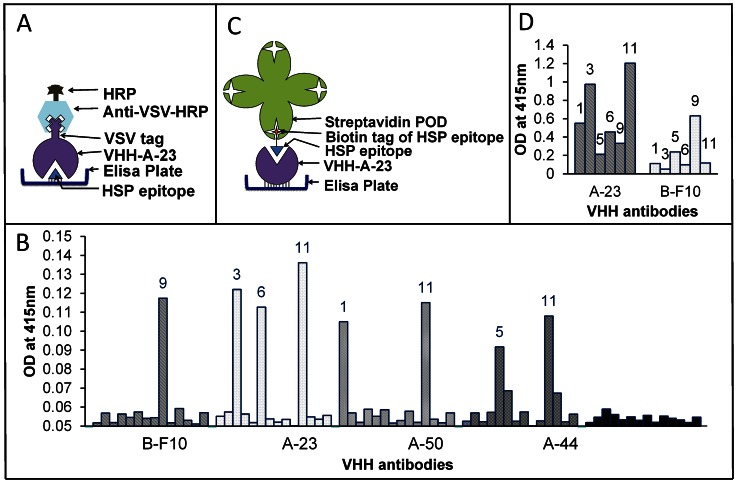
Peptides (14 overlapping 20-mers) representing potential epitopes of the HSP protein mapped against different VHH antibodies in direct ELISA. Panel A - Scheme applied in B; Panel B - Results of direct ELISA with coated peptides; Panel C - Scheme applied in D; Panel D - Direct ELISA with VHH antibodies coated to the well.

The interactions of fourteen overlapping linear 20-mer peptides (Figure 1), based on the 144 amino acid long HSP, with 9 VHH antibodies were first studied using linear peptides as capture species (Figure 2A, 2C). For each antibody binding peptides were found. Antibody A-23 bound to peptides #3, #6 and #11, antibody A-50 bound to #1 and #11, whereas B-F10 bound only to #9 (Figure 2B). The summary of interactions of all epitopes with all 9 VHH antibodies is given in Table 1. Overall it was found that as many as 7 out of 14 peptides were recognized by 8 out of 9 different VHH antibodies as summarized in Figure 1 and Table 1. The binding of antibodies can be grouped in three categories. Group 1 consists of antibodies binding to three different epitopes (A-23), group 2 those binding to two different epitopes (A-44, A-50, A-89 and B-A1), and group 3 those binding to a single epitope (B-D8, D-F9 and B-F10). Interestingly, all antibodies positively selected on purified antigen by a direct phage display selection procedure (represented here with prefix A) recognized peptide #11, whereas most of the ones negatively selected via a depletion strategy (prefix B) recognized other epitopes, which may therefore be more selective. To verify that the observed interactions were not artifacts of the peptide immobilization method we also carried out a reverse analysis using two VHH antibodies (A-23, B-F10) with the highest binding affinity constants as capture species [18]. For this purpose the peptides #1, #3, #5, #6, #9, and #11 were biotin-tagged and assayed as in Figure 1C. The results in Figure 1D confirmed that A-23 binds peptides #3 and #11, while binding to epitope #6 was at background level, and not conclusive. B-F10 was confirmed to only bind to peptide #9.

**Table 1:** Summary of interactions of 14 peptides derived from the HSP protein with 9 different anti-HSP VHH antibodies [18].

VHH Antibody	Response to different epitopes (#1 to #14) of 16 kDa HSP from tuberculosis													
	#1	#2	#3	#4	#5	#6	#7	#8	#9	#10	#11	#12	#13	#14
A-23			X			X					X			
A-44					X						X			
A-50	X										X			
A-89				X							X			
B-A1				X							X			
B-B12														
B-D8			X											
B-F9			X											
B-F10									X					

#### B. Epitope mapping using HSP in sandwich ELISAs and sandwich SPR measurements

**Figure 3:**
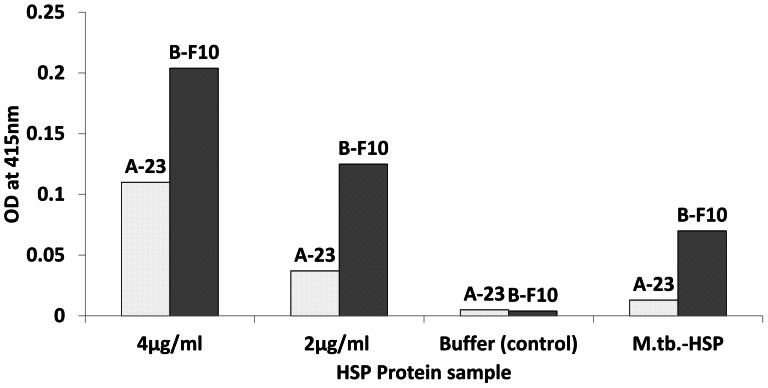
Comparison of purified recombinant HSP protein with *M.tb*.-HSP in sandwich ELISA assays with antibody A-23 AVI as capture antibody.

**Figure 4:**
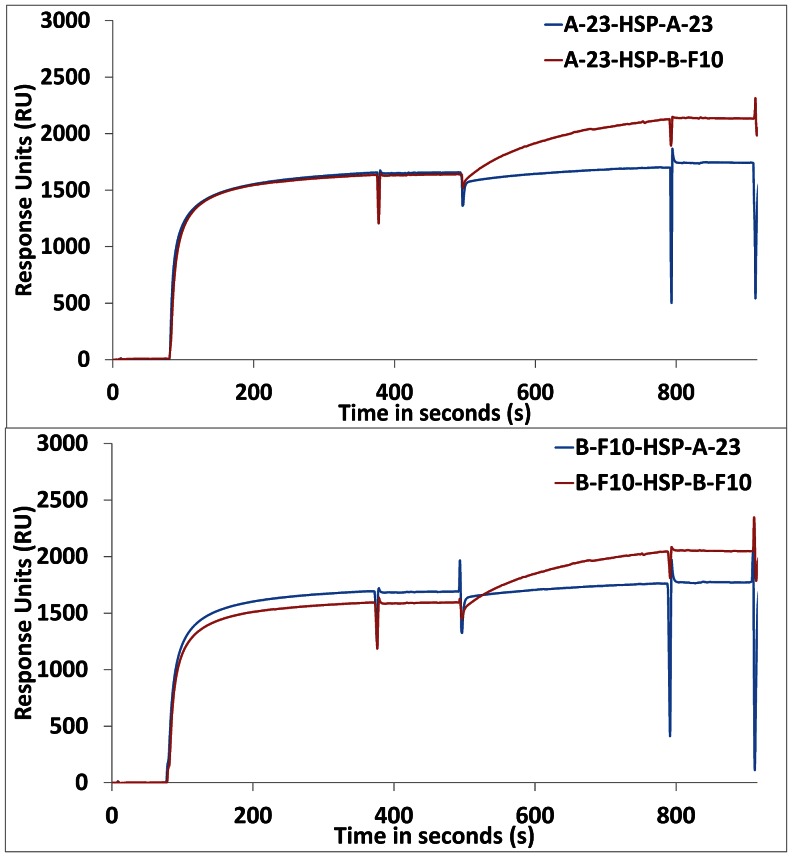
Plots of “sandwich SPR” showing the binding kinetics of B-F10 or A-23 binding to HSP as a secondary antibody in a sandwich assay in which HSP was first captured by A-23 or B-F10. Four different sandwich variations are shown: A-23-HSP-A-23 or -B-F10 (top panel) and B-F10-HSP-A-23 or -B-F10 (lower panel).

If HSP would behave as a protein monomer then a sandwich ELISA using the same VHH for both capture and detection would not result in a signal, whereas the use of two VHH recognizing distinct epitopes that do not interfere sterically would yield a signal. To test this a sandwich ELISA with rHSP-tag protein captured by A-23-AVI was carried out using the two most potent and distinct VHH antibodies B-F10-VSV and A-23-VSV for detection. In Figure 3, it is shown that rHSP-tag and *M.tb.-*HSP protein when captured by A-23, could be detected by both secondary antibodies i.e. A-23 as well as B-F10. The A-23 detection suggested that both recombinant HSP and *M.tb.*-HSP do not behave as monomers. For *M.tb.-*HSP, the signal was lower than for 2 µg/ml recombinant HSP protein, presumably due to the lower concentration of the HSP protein in the *M.tb.-*HSP. At lower concentrations, the VHH antibody B-F10 yielded 3–4 times higher signals compared to A-23 for both the recombinant HSP protein as well as the HSP in *M.tb.-*HSP. The basis of this difference could be related to the difference in binding constants of the VHH antibodies (i.e. B-F10 (K_d_ = 0.4×10^−9^ M) is more efficient than A-23 (K_d_ = 2.4×10^−9^ M)), but also to the reported native higher order (dodecameric, [29]) protein structure in relation to the specific epitopes recognized. To disentangle kinetic, conformational and steric effects, further analysis by “sandwich-SPR” was carried out. A-23 or B-F10 were used to capture recombinant HSP followed by either A-23 or B-F10 as detecting antibody (Figure 4 and Table 2). With A-23 as capture antibody, B-F10 showed much higher binding to HSP (703 RU) compared to A-23 (172 RU). This confirmed the peptide study that A-23 and B-F10 bind to distinct epitopes and would support a model of a protein monomer. However, with B-F10 as capture antibody, again B-F10 showed much higher binding (609 RU) compared to A-23 (168 RU). This contradicted a monomer model for recombinant HSP and called for a more complex multimeric model of HSP.

**Table 2:** SPR assay results showing the observed response units (RU) for rHSP-tag (HSP) and the detection antibodies A-23 and B-F10.

#	Capture/ detection Antibody combinations	*HSP (RU)*	*Detection* *Antibody (RU)*	*Observed efficiency*			
				*Monomer model*	*Dimer model*	*Trimer model*	*Dodecamer model*
1	A-23 / HSP / A-23	2664	172	not possible	15%	11%	8%
2	B-F10 / HSP / A-23	2290	168	9%	17%	13%	9%
3	A-23 / HSP / B-F10	2028	703	40%	82%	61%	45%
4	B-F10 / HSP / B-F10	2020	609	not possible	71%	53%	39%

These responses are interpreted in terms of secondary binding efficiencies relative to different models of HSP oligomerization.

### Size Exclusion Chromatography (SEC) to Determine the Oligomeric Nature of HSP

**Figure 5:**
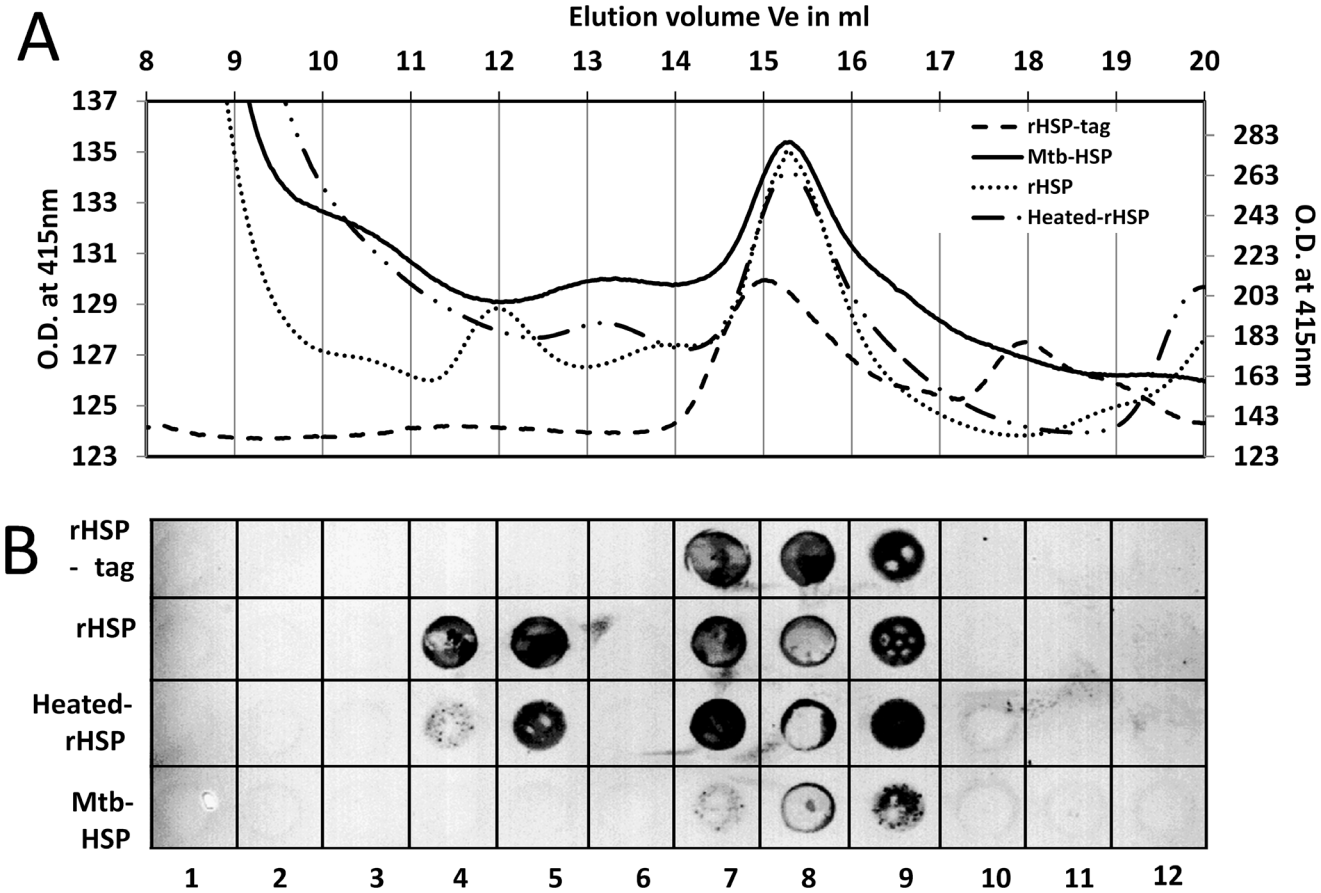
Size exclusion chromatography (SEC) for apparent molecular weight determination of HSP. Where figure A shows SEC plot of different HSP samples i.e. purified recombinant HSP with tag (rHSP-tag), unpurified recombinant native HSP ( rHSP), heated unpurified recombinant native HSP ( heated-rHSP) and *M.tb.* lysates (Mtb-HSP) while figure B shows the dot blot of interaction of 16 kDa protein present in these SEC fractions with antibody B-F10, where lane 4&5 shows fractions of different HSP protein eluting at ∼301 kDa, lane 7–9 shows fractions of different HSP protein eluting at ∼55–61 kDa.

In the literature, it is reported that the HSP protein exists either as monomer [30], [31], dimer [30], [31], trimer of trimers [31], [32] or dodecamer [29] depending upon the method of extraction from *Mycobacterium* or *E. coli*. To obtain an indication of the quaternary structure of recombinant and native HSP protein after standard isolation procedures, SEC was performed on four samples, i.e. (1) purified recombinant HSP with an AVI and HIS tag on the C-terminus (rHSP-tag) refolded from urea-dissolved inclusion bodies in *E.coli*, (2) French press generated lysate of native recombinantly HSP protein without tags expressed in *E.coli (rHSP),* (3) heat inactivated French press generated lysate of native recombinant HSP protein without tags expressed in *E.coli (Heated-rHSP),* and (4) heat-inactivated *M.tb.* lysates generated by mechanical shear with zirconium beads also containing HSP (Mtb-HSP) [2]. In Figure 5A the elution profiles obtained from each sample are super-imposed. rHSP-tag protein has a monomer size of 20.9 kDa due to the two (AVI and HIS) tags. On SEC, purified protein separated into two peaks with sizes corresponding to 14±5 and 61±20 kDa. Unpurified rHSP with HSP as major component also showed two peaks at 301±97 kDa and 55±18 kDa. After heating heated-rHSP, only the peak at 55±18 kDa remained. Unpurified heated *M.tb.*-HSP also showed only one peak at 55±18 kDa. The presence of HSP protein in samples from these respective fractions was determined using dot blot analysis (Figure 5B). Major peaks (fractions 7–9) eluting at ∼55–61 kDa (33% error margin) all contained HSP protein reactive to A-23 and B-F10, but peak (fraction 10–11) eluting at 14±5 kDa did not. Native rHSP protein peak (fraction 4–5) eluting at ∼301 kDa also contained the HSP protein. In case of heated-rHSP the fraction 4–5 eluting at ∼301 kDa contained much less HSP compared to non-heated rHSP. This shows that French press generated *E. coli* lysate of rHSP which avoids heating, mostly leaves rHSP in higher eluting (301 kDa) conformation whereas in the case of heated-rHSP it is mostly found to be eluting at ∼55–61 kDa. This is also evidence that indeed *M.tb.*-HSP, rHSP, heated-rHSP and rHSP-tag behave like multimers of different sizes and do not occur as monomers.

### ESI-TOF-MS to Determine Oligomeric Nature of HSP

**Figure 6:**
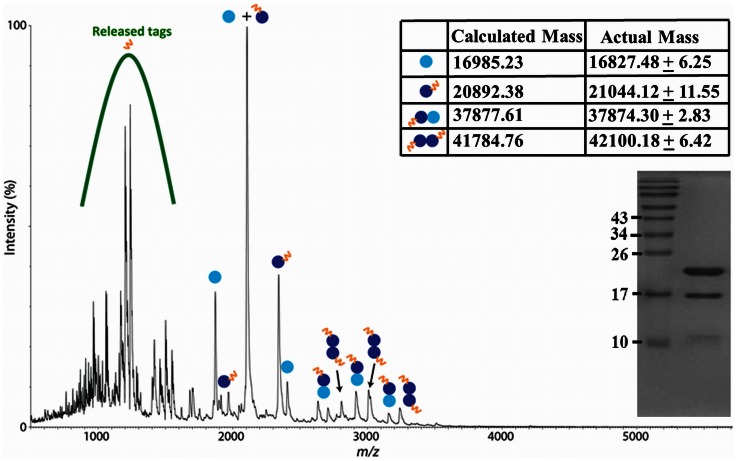
Native ESI-TOF-MS spectrum of HSP confirming absence of oligomers greater than the dimer after urea-based isolation. Sample complexity at low *m/z* is due to proteolytic loss of tags prior to purification. The various HSP species are indicated in the spectrum: monomer without tag (light blue circle), monomer with tag (dark blue circle), dimer formed from monomers with tags (double dark blue circles), and dimer formed from mixed monomers (double circles, light and dark blue). Other signals in the spectrum stem from contaminants, including the released tags. Deviations of calculated and measured mass values are by exopeptidases pruning the proteins. Denaturing SDS-PAGE coomassie stained gel (inset) shows two prominent bands at 21 and 17 kDa size corresponding to monomers with and without tags.

Electrospray ionization time-of-flight mass spectrometry (ESI-TOF-MS) is a highly accurate method to determine the quaternary structure of complexes of proteins compared to SEC [33]. A nanoelectrospray ionization time-of-flight (nESI-TOF) analyzer was used for this mass spectrometric analysis of rHSP-tag under native conditions. The use of a volatile buffer (ammonium acetate) permitted the retention of non-covalent interactions in the gas phase, such that accurate masses of protein complexes could be obtained. The sample of rHSP-tag that was analyzed is shown on an SDS PAGE gel (inset Figure 6). A dominant band of 21 kDa (rHSP-tag) and a less intense band of 17 kDa (rHSP without tag) were observed plus less defined low molecular weight molecules. The nESI-TOF mass spectrum (Figure 6) shows the presence of these two monomer masses, in addition to peaks corresponding to homo- and hetero-dimers of the two monomers. Interestingly, the homodimer of rHSP without tag was not found, presumably because it was not purified on the His-tag column. The small differences between calculated and observed molecular masses could be due to proteolytic trimming of the protein. The two monomeric species we assume to be derived from the dimers, but to be less stable under the conditions of the ammonium acetate buffer in combination with the nESI-TOF conditions. No oligomers larger than the dimer were observed, confirming that standard HSP isolation procedures, where HSP is refolded from urea-dissolved inclusion bodies in *E.coli*, reduces the HSP from the native dodecameric form to a dimeric form.

## Discussion

In this study, the binding of a set of different VHH antibodies to the 16 kDa HSP was probed with the help of different techniques (ELISA, SPR, and SEC) using both peptides and whole protein. The results demonstrated that after mechanical release from heat-killed bacteria HSP behaves as a dimer. Optimal combinations of antibodies were selected for the development of diagnostic sandwich assays.

Tuberculosis is a contagious disease so patient samples are usually heat-inactivated at 80°C for 30 mins, before performing any diagnostic test. The heat-killing step was also used in the original procedure to generate *M.tb.* specific antibodies by injecting llamas with heat-killed lysates of *M.tb.* [18]. However, such procedures normally tend to effectively denature most proteins, so that the immunization procedure was likely selective for heat-tolerant proteins. All selected VHH were reactive only with HSP as demonstrated by Trilling *et. al.* [18]. This bias for binding of the 16 kDa HSP may, therefore, be the result of the standard heating practice. The heat tolerance adds to HSP being a robust protein marker [18]. Knowledge of the exact conformation of native HSP after applying denaturing conditions by this procedure is necessary for the development of reliable immunological diagnostic procedures that are strongly dependent on protein conformation in specific sandwich assays.

Previous studies have shown immune-response to epitopes #3, #8, #10 and #12 with human T cells [34], epitope #3, #4, #6, #7, #8 and #13 for mouse Mabs, and epitopes #8, and #9 for human B cells [22]. We show using ELISA based on llama monoclonals instead of sera that 7 out of 14 epitopes were recognized by 8 out of 9 different llama VHH antibodies, and that epitopes #3, #4, #6, #11 and #13 were the epitopes recognized most. Immunodominance of epitopes #3, #4, #6 and #13 was shown previously for human sera after natural infection, but epitope #11 emerged as a new target in llama immunizations performed with heated lysates as described above by Trilling *et.al.* [*18*]. Epitope #11 was the most frequently recognized epitope, showing interactions with five different antibodies, although frequencies of phage display selected antibodies may have no direct relationship to dominance of these antibodies in llama serum. Antibodies like B-D8, B-F9, B-F10 that bound to only one peptide, indicated that their recognition sites lie in the center of the peptide, which in the 3D structure was either a loop or the coil region of the protein (Figure S1). The overlapping adjacent epitopes may lack the full binding surface as it only represents half of the loop or coil. Several antibodies recognized more than one epitope. Interaction with multiple epitope domains of HSP could be due to a complex recognition site composed of several proximal peptide loops.

In the literature, for HSP, monomer [30], dimer [31], trimer [32], trimer of trimers (nonamers) [32] or dodecamer structures [29] have been described. The monomer and dimer configurations were based on reverse-phase high-performance liquid chromatography (HPLC), and on HSP protein purified by gel filtration chromatography [30], [31]. Trimer and nonamer configurations were based on cryo-electron microscopy [32], whereas the dodecamer configuration was deduced from SEC analysis [29]. These determinations were either based on native or denatured proteins, but for native protein only the SEC analysis has been reliable for size determination. Our SEC analysis initially suggested that HSP (16 kDa) occurred both as a trimer (16×3 i.e. 48 kDa) and a hexamer of trimers (18-mer 16×18 i.e. 288 kDa) with observed sizes of 55 kDa and 301 kDa. However, within the SEC margin of error, HSP could also be a dimer and a hexamer of dimers (dodecamer) as reported before [29] and with expected sizes of 32 kDa and 192 kDa respectively. The slightly larger observed size (61 kDa) of the putative dimer of rHSP-tag compared to the putative dimers of the native rHSP, heated-rHSP and *M.tb.-*HSP proteins with a size of 55 kDa is consistent with the presence of the tags (2×5 kDa). To resolve the issue we analyzed the oligomeric structure by ESI-TOF-MS and concluded that the rHSP-tag sample contains dimers, but not trimers.

In our sandwich ELISA and SPR experiments the immunological reactivity of HSP in SPR did not fit a monomer model, whereas a dimer model resulted in the best fit of our data. This can be seen from Table 2 where we calculated the observed efficiency of binding of the secondary, “sandwich” antibody A-23 and B-F10 relative to the theoretical maximum efficiency in relation to different possible configurations of HSP as a monomer, dimer, trimer or dodecamer using a the following two formulas:
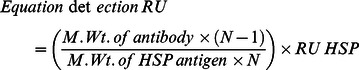


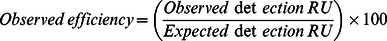
Where N represents the degree of multimerization. N-1 is used because the HSP antigen is assumed to be captured first by one antibody.

If HSP were a monomer, A-23-HSP-A-23 or B-F10-HSP-B-F10 sandwiching would not be possible. B-F10-HSP-B-F10 (609 RU), for which only one epitope was known, showed strong secondary binding, however. If the HSP would be a dodecamer then in theory each of the 12 captured HSP units might bind one secondary antibody each except for the anchoring unit. Given the dodecamer model for A-23-HSP-B-F10 we only observe 45% actual secondary binding, but given the dimer model 82 % secondary binding is observed.

**Figure 7:**
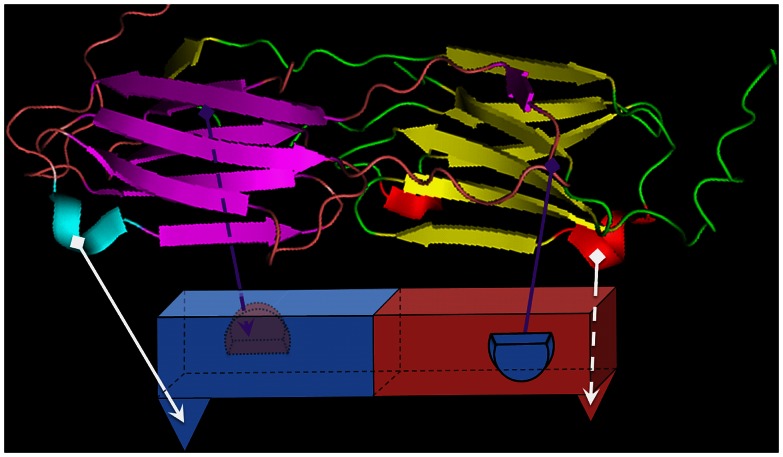
Model showing the arrangement of dimer structure of HSP protein along with the representation of possible sites of VHH antibodies B-F10 and A-23.

An unexpected result of the SPR experiments was that, in contrast to B-F10, A-23 would not bind efficiently to A-23- or B-F10-captured HSP. We think that this was not related to the affinity of A-23 for HSP, because A-23 captured similar amounts of HSP from the solution as B-F10 in the same time period. Rather we think that there are steric reasons why A-23 and B-F10 are behaving so differently. To understand this steric interference it was necessary to map the information on the epitopes recognized by both antibodies i.e. A-23 and B-F10 on to the proposed 3D structure model of HSP (Figure 7) [29]. To B-F10 only epitope #9 would bind, whereas to A-23 two peptides #3 and #11 were consistently binding. Epitope #3 sites for A-23 are not part of the 3D model, as the dodecameric structure deduced by Kennaway *et.al.* does not show its arrangement [29]. We found that the dodecameric structure proposed for HSP by Kennaway *et.al.* was built up from 6 dimers, and that the formation of the higher multimeric structure is dependent on interactions of all C-termini with adjacent protein dimers. Dimers are, therefore, the basic conformational unit of HSP, which might survive heat denaturation. Interestingly then, in the dimer 3D model the two positions of epitope #11 of antibody A-23 appear to be in the same plane, whereas the two positions of epitope #9 of antibody B-F10 are diagonally arranged to each other in opposite planes (Figure 7).

**Figure 8:**
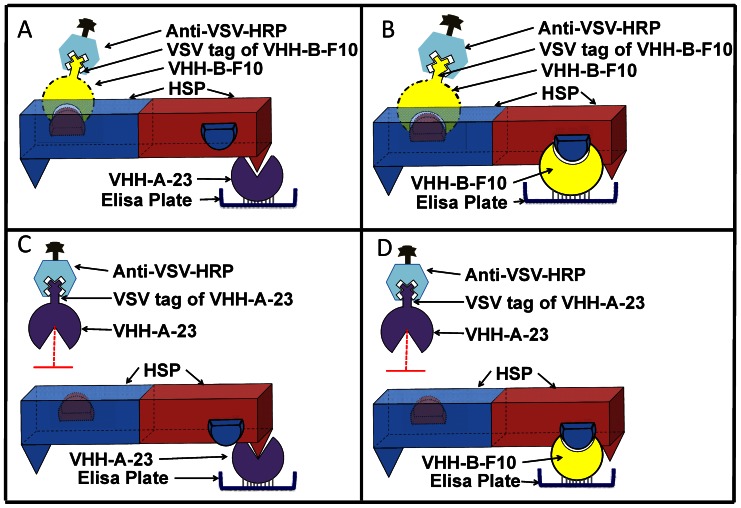
Models explaining the blocked detection by A-23 antibodies. The diagrams show the capture of HSP dimer with VHH antibodies B-F10 and A-23 and successful detection with B-F10 panels A–B) and failed detection with A-23 (panels C–D).

Based on the accumulated evidence from SEC, ESI-TOF-MS, SPR, and the 3D structure of the HSP protein, a model is proposed which shows schematically how B-F10 and A-23 bind to the HSP dimer (Figure 8). As per this dimer model, all binding sites for A-23 are in the same plane, and thus, upon capture by antibody A-23, the other A-23 binding site is blocked. Binding of VHH antibody B-F10 is still possible as that binding site is perpendicular to the site of A-23, in a different plane. By contrast, if antibody B-F10 is used for capturing, the binding sites of B-F10 are diagonally arranged in opposite planes, allowing strong binding, while apparently A-23 binding sites remain unaccessible.

In conclusion, the different VHH antibodies showed specific binding to many different epitopes of the 16 kDa HSP. The VHH’s B-F10 and A-23 were the best binders of HSP and are complementary to each other as they recognize non-overlapping epitopes on the HSP dimer. ESI-TOF-MS and SEC in combination with immuno-dot blot analysis showed that although the protein exists as a dodecamer in native state, the HSP is a dimer both as recombinant AVI-HIS-tagged protein (rHSP-tag) and in heat-inactivated *M.tb.* lysates (*M.tb.-*HSP and heated-rHSP). Based on these findings, assays for protein-based diagnosis of tuberculosis could be developed. High concentrations of this non-secreted can be obtained by first concentrating the *M.tb.* cells from liquefied sputum samples using magnetic TB-Beads [35], followed by controlled lysis of the bacteria using mechanical or ultrasonic [36] lysis methods.

## Supporting Information

Figure S1 **Arrangement of odd and even epitopes on the 16 kDa heat shock protein from** ***M.Tb*** **.**
(TIF)

Figure S2 **Plot for calibration of Superdex 200 10/300 GL SEC column with known standards.**
(TIF)

Table S1 **Comparison between the actual and estimated molecular weights of the standard proteins along with the deviation percentage while calibrating the SEC column.**
(TIF)
