# Supplementary figures and images for: 16 kDa Heat Shock Protein from Heat-Inactivated Mycobacterium tuberculosis Is a Homodimer – Suitability for Diagnostic Applications with Specific Llama VHH Monoclonals
Source: PLoS One. 2013 May 30;8(5):e64040. doi: 10.1371/journal.pone.0064040 (PMC3667823; doi:10.1371/journal.pone.0064040)

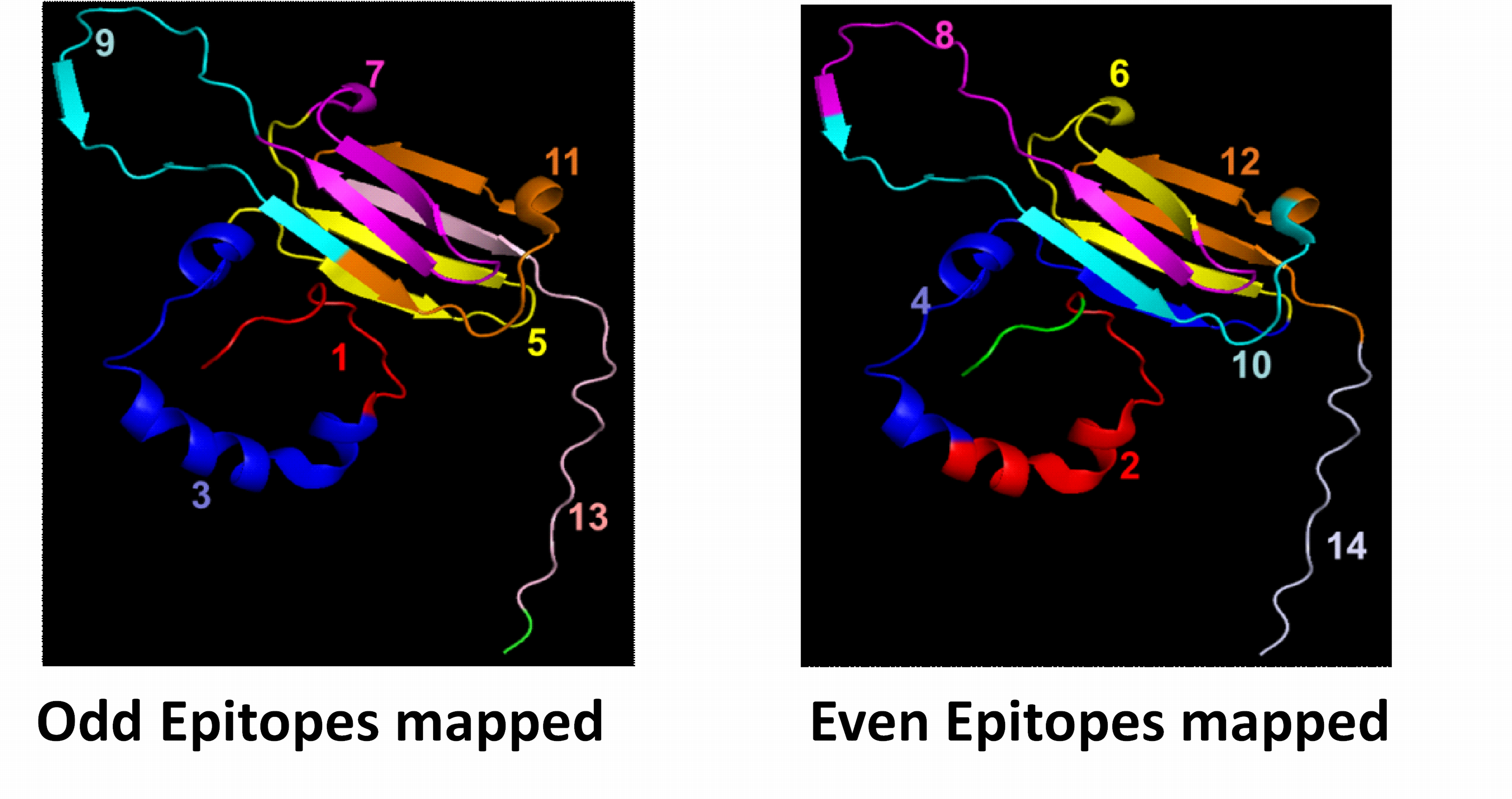

Supplement: Figure S1 — Arrangement of odd and even epitopes on the 16 kDa heat shock protein from M.Tb . (TIF) [file pone.0064040.s001.tif]

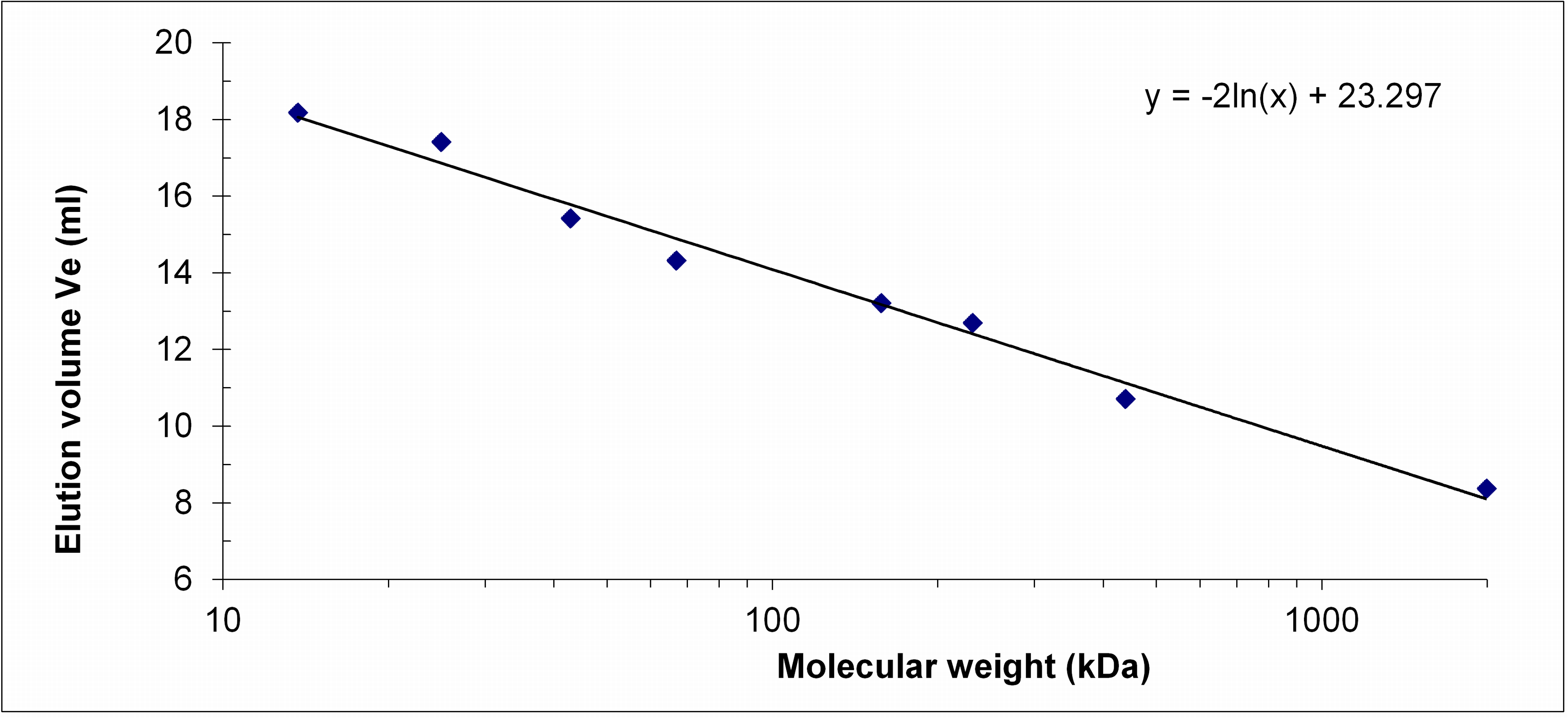

Supplement: Figure S2 — Plot for calibration of Superdex 200 10/300 GL SEC column with known standards. (TIF) [file pone.0064040.s002.tif]

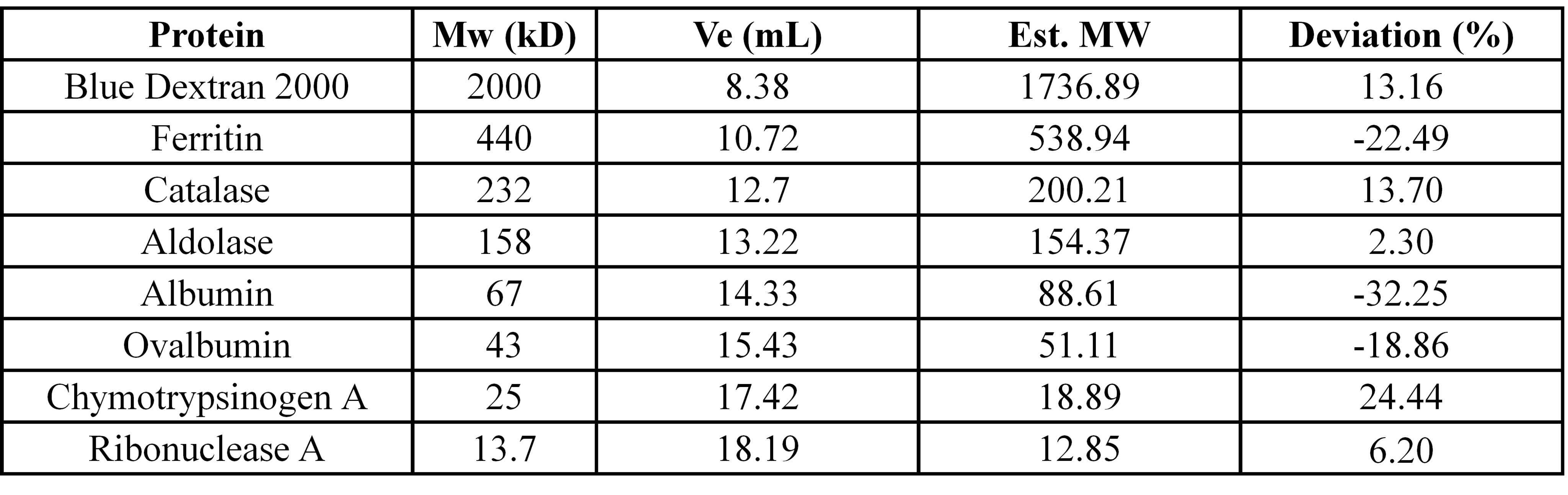

Supplement: Table S1 — Comparison between the actual and estimated molecular weights of the standard proteins along with the deviation percentage while calibrating the SEC column. (TIF) [file pone.0064040.s003.tif]
